# Supplementary material for: Functional investigation of a putative calcium-binding site involved in the inhibition of inositol 1,4,5-trisphosphate receptor activity
Source: J Biol Chem. 2025 Feb 11;301(3):108302. doi: 10.1016/j.jbc.2025.108302 (PMC11938044; doi:10.1016/j.jbc.2025.108302)
Supplement: Supplementary Figure 1 [file mmc1.pdf]

A

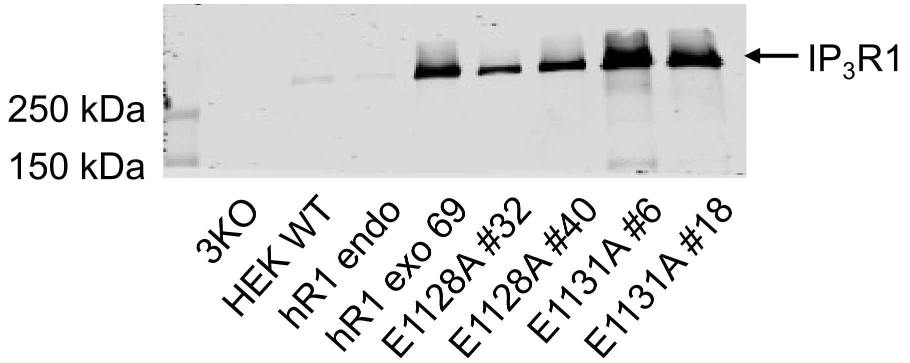

B

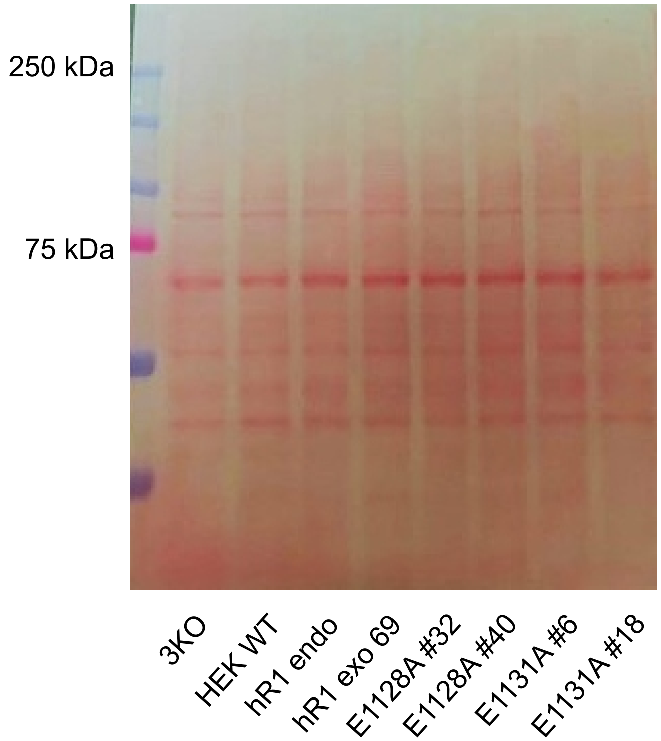

Supplemental Figure 1

Supplemental Fig. 1. In A. Overexposed image of the western blot shown in Fig 2. to visualize endogenous levels in comparison to stable cell lines expressing the indicated constructs. B shows the ponceau stained membrane to indicate protein loading levels prior to immunoblotting.
